# Self-directed arm therapy at home after stroke with a sensor-based virtual reality training system

**DOI:** 10.1186/s12984-016-0182-1

**Published:** 2016-08-11

**Authors:** Frieder Wittmann, Jeremia P. Held, Olivier Lambercy, Michelle L. Starkey, Armin Curt, Raphael Höver, Roger Gassert, Andreas R. Luft, Roman R. Gonzenbach

**Affiliations:** 1 Rehabilitation Engineering Laboratory, Department of Health Sciences and Technology, ETH Zurich, Switzerland; 2 Division of Vascular Neurology and Rehabilitation, Department of Neurology, University Hospital Zurich, Zurich, Switzerland; 3 Hocoma AG, Volketswil, Switzerland; 4 Spinal Cord Injury Center, Balgrist University Hospital, Zurich, Switzerland; 5 Cereneo Center for Neurology and Rehabilitation, Vitznau, Switzerland

**Keywords:** Rehabilitation, Stroke, Feasibility, Arm, Virtual reality therapy, Video games

## Abstract

**Background:**

The effect of rehabilitative training after stroke is dose-dependent. Out-patient rehabilitation training is often limited by transport logistics, financial resources and a lack of motivation/compliance. We studied the feasibility of an unsupervised arm therapy for self-directed rehabilitation therapy in patients’ homes.

**Methods:**

An open-label, single group study involving eleven patients with hemiparesis due to stroke (27 ± 31.5 months post-stroke) was conducted. The patients trained with an inertial measurement unit (IMU)-based virtual reality system (ArmeoSenso) in their homes for six weeks. The self-selected dose of training with ArmeoSenso was the principal outcome measure whereas the Fugl-Meyer Assessment of the upper extremity (FMA-UE), the Wolf Motor Function Test (WMFT) and IMU-derived kinematic metrics were used to assess arm function, training intensity and trunk movement. Repeated measures one-way ANOVAs were used to assess differences in training duration and clinical scores over time.

**Results:**

All subjects were able to use the system independently in their homes and no safety issues were reported. Patients trained on 26.5 ± 11.5 days out of 42 days for a duration of 137 ± 120 min per week. The weekly training duration did not change over the course of six weeks (*p* = 0.146). The arm function of these patients improved significantly by 4.1 points (*p* = 0.003) in the FMA-UE. Changes in the WMFT were not significant (*p* = 0.552). ArmeoSenso based metrics showed an improvement in arm function, a high number of reaching movements (387 per session), and minimal compensatory movements of the trunk while training.

**Conclusions:**

Self-directed home therapy with an IMU-based home therapy system is safe and can provide a high dose of rehabilitative therapy. The assessments integrated into the system allow daily therapy monitoring, difficulty adaptation and detection of maladaptive motor patterns such as trunk movements during reaching.

**Trial registration:**

Unique identifier: NCT02098135.

## Background

Functional outcome following stroke is positively correlated with the dose of the applied rehabilitative intervention [1]. Therefore, post-stroke therapy should be provided at a high intensity, a high frequency and over long periods of time [1, 2]. However, the delivery of intensive physical therapy requires extensive therapist support, is expensive, and is often limited by the low compliance and lack of motivation to perform rehabilitative training at the recommended frequency [3]. This can lead to functional deterioration, e.g., by learned non-use of the affected limb [4].

Self-directed home therapy, supported by dedicated instrumented devices [5–7] or virtual reality gaming platforms [8–13], could help to increase the dose of rehabilitation at low cost without the need for direct supervision by a therapist. It is important that such home therapy adapts to changes in the subject’s performance in order for it to remain challenging and motivating [8]. On the other hand, unsupervised rehabilitative training could lead to inefficient or harmful (i.e. maladaptive) movement sequences or pain, and could potentially worsen performance [8, 11, 14]. Home therapy should, therefore, include monitoring of movement quantity and quality. Several platforms dedicated to upper-extremity home rehabilitation have been proposed [6, 7, 15–17]. However, to the best of our knowledge only few were actually installed in the patients' homes for several weeks and tested for feasibility beyond case studies. These home studies always involved some external supervision, in the form of e.g. on-site visits [16, 17], tele-monitoring and adaption [16, 17] or telephone calls [6, 7], which might have affected compliance and motivation and thereby therapy dosage. However, such an approach requires manpower, which limits the affordability and scalability of home-based therapy. The feasibility and compliance of completely unsupervised upper-limb stroke therapy over the course of several weeks remains to be investigated.

In this paper we investigate the feasibility of self-directed home training with the custom-designed ArmeoSenso system [18], a virtual reality arm rehabilitation platform based on wearable inertial measurement units (IMU). In a clinical study involving eleven patients with hemiparesis of the arm due to stroke, we evaluated the ability to deliver therapy at a high dose through simple-to-use and entertaining, yet functionally relevant and adaptive rehabilitation games. Unsupervised, automated assessments integrated into each therapy session allowed monitoring of arm function, and detection of undesired compensatory movements.

## Methods

### ArmeoSenso training system

**Fig. 1:**
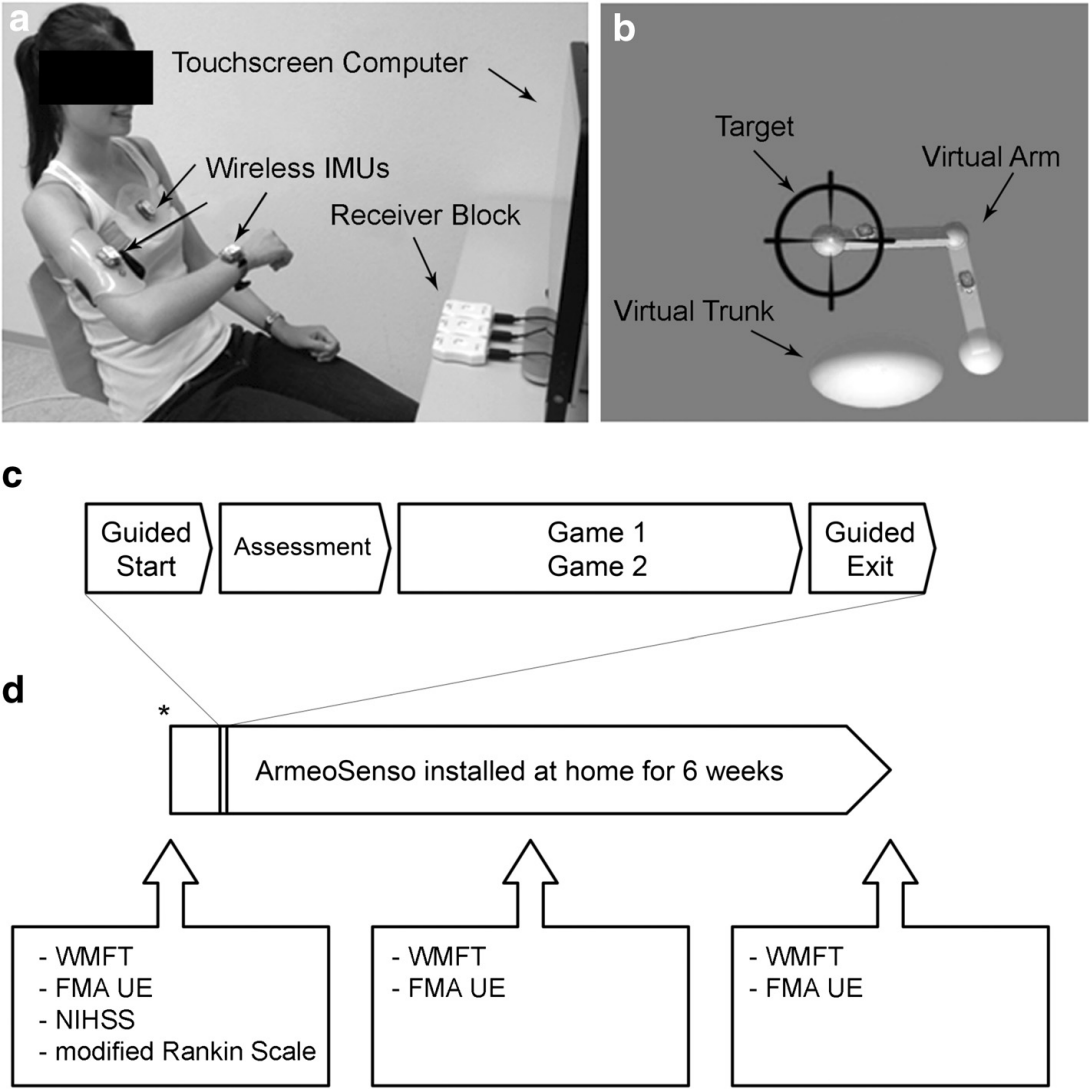
System Overview and Study Outline. **a**: Photograph of a healthy subject using ArmeoSenso. **b**: Screenshot of the pointing task assessment: the virtual upper- and lower arm and the trunk are displayed. The arm points to a target. **c**: Sequence of a training session. Before each training session, two automated assessments are performed. **d**: Study outline: The ArmeoSenso system is installed in the patient's home for six weeks. The patients are assessed clinically before the start, after three weeks, and after six weeks of training. Abbreviations: WMFT: Wolf Motor Function Test; FMA-UE: Fugl-Meyer Assessment Upper Extremity; NIHSS: National Institute of Health Stroke Scale. *system installation and patient instruction by a therapist

ArmeoSenso comprises a motion capture system based on wearable sensors in combination with an all-in-one touch screen computer (Inspiron 2330, Dell Inc., Fig. 1a). The therapy software provides a user-friendly graphical user interface, two therapy games, and two short automated assessments of arm function [18]. For real-time tracking of arm and trunk movements, the patient wears three IMUs (MotionPod 3, Movea Inc.) fixed to the lower and upper arm as well as the trunk (Fig. 1a). The IMUs measure acceleration, angular velocity and the magnetic field, all in three dimensions, and stream this data wirelessly to a receiver block, which is connected to the computer via USB and serves as a docking station to charge the sensors. A kinematic reconstruction estimates the orientation of the trunk, the upper- and the lower arm based on the Madgwick algorithm [19] and the corresponding joint positions are computed with forward kinematics [20]. This reconstruction serves as input for the assessments and therapeutic virtual reality games (Fig. 1b). By using the same virtual kinematic parameters for each patient, virtual sizes, e.g. distances or the size of targets, are normalized to the patient’s body size. To discourage trunk inclination or rotation during pointing movements, the arm movements are computed and displayed relative to the trunk.

### Sequence of a training session

A typical training session is illustrated in Fig. 1c. The patient uses the unaffected hand to touch a start button on the screen, which triggers visual instructions on how to remove the IMUs from the receiver block, don them on and perform a simple calibration procedure (i.e. guided start). For the calibration, the patient has to sit upright and hold the impaired arm in a horizontal position directed towards the screen for five seconds to determine the orientation of the IMUs on the patient's body. For calibration, the patient was allowed to use the unaffected arm for support.

Automated unsupervised assessments, conducted before every therapy session, evaluate arm function on two standardized tasks that remained identical throughout the therapy. The first was a pointing task which aims to evaluate the ability and time required for reaching a virtual target. The targets appear consecutively and in random order at nine pre-defined target positions located within the reachable workspace of a healthy individual. The patients are instructed to reach the target as quickly as possible and then stay on the target for two seconds. If a target is not reached within eight seconds, it disappears and a penalty time of eight seconds is taken instead. The number of targets reached and the mean time to reach the targets are reported. Joint angles are recorded to detect maladaptive compensatory movements such as excessive trunk inclination or trunk rotation during reaching.

The second assessment measured the two-dimensional workspace of the impaired arm in the transverse plane. Patients are instructed to actively reach out as far as possible with their impaired arm and to explore the entire arm workspace, similar to previous studies [21]. The attained workspace is displayed and computed as the number of squares of ten centimeters side length arranged in a transverse plane relative to the patient’s trunk.

Therapy games: The aim of the therapy game ‘Meteors’, was to improve arm workspace and reaching velocity. In this game scenario, a virtual arm which matches the movement of the patient’s arm is used to catch meteors that fall towards a planet. In contrast, the aim of the therapy game ‘Slingshot’ was to train arm coordination and to improve the precision of arm pointing and reaching movements. The patient holds a virtual slingshot to shoot stones at static or moving targets of variable size by pointing at the target with the slingshot and extending the elbow according to the target, which requires both precision and endurance. In both games, a performance-based (i.e. speed, number of targets reached, etc.) score is computed and used to dynamically adapt the difficulty of the game (e.g. meteors and targets move faster, or appear smaller etc.) in order to keep motivation and engagement high. The targets are placed within or at the border of the patient's 3D workspace, which is continuously estimated with a voxel-based model, to keep the challenge high, promote an increase in arm workspace, and prevent frustration [18].

### Study design

The study was designed as an open-label, single group clinical trial to study the feasibility and safety of performing arm rehabilitation with the ArmeoSenso system in the patient’s home without any supervision. Inclusion criteria were a minimum age of 18 years, hemiparesis of the arm due to cerebrovascular ischemia, the ability to lift the paretic arm against gravity, a minimal arm workspace of 20 cm x 20 cm in the horizontal plane and absence of aphasia, depression, dementia and hemianopia. ArmeoSenso was installed on a table, and instructions for proper usage were given by a trained physiotherapist to the patient, prior to the start of the study. No modification of the patient’s house was required. Patients were asked to use the system as often as possible over a period of six weeks. They decided by themselves about the training duration and frequency and could start or stop a therapy session at any time. The patients' usual therapy continued and was not altered during the study. A structured patient interview was conducted at the end of the trial. The study followed GCP-guidelines and was approved by the local Cantonal ethics committee Zurich (KEK-ZH: 2013–0182) and the Swissmedic (2013-MD-0019). All subjects gave written informed consent in accordance with the declaration of Helsinki.

### Outcome measures

The primary safety outcome was any adverse event related to the system that occurred during the study period. The primary outcome of the study was the duration of training per week with ArmeoSenso. This was used as an indicator of therapy acceptance and feasibility of unsupervised therapy. As a measure of motivation, we investigated whether system usage changed over time. We report the average training duration for every training week, the training duration per session (equal to the minutes of playing games per ArmeoSenso session) and the training frequency (equal to the number of days of ArmeoSenso usage). The sum of both the number of meteors caught (Meteors game) and the number of targets hit (Slingshot game) was used as a measure of training intensity. To assess the efficiency of training, we quantified the training duration in relation to the overall time spent with the system, which includes the time for automated assessments and for system setup.

To investigate whether patients compensated for their arm impairment by moving their trunk, we analyzed trunk rotation and inclination during successful pointing movements in the pointing task assessment for one target. Trunk rotation and inclination were recorded at onset (initiation) of the movement and once the hand reached the target (final). The respective absolute difference between initial and final trunk orientation was treated as the patient's trunk compensation. The subtraction also serves to remove bias, e.g. due to sensor misalignment or magnetic field disturbances [22, 23], while the short duration of 8 s or less should minimize effects of orientation drift [24], e.g. due to gyroscope bias. As a control, patients performed the same task with their unaffected arm 10 consecutive times within one session at the end of the home trial.

Arm function was assessed clinically using the Fugl-Meyer Assessment - Upper Extremity (FMA-UE) [25] and the Wolf Motor Function Test (WMFT) [26] at 3 time points (see Fig. 1d) and with ArmeoSenso-based automated assessments, as described above.

### Statistical methods

Descriptive statistics are reported as mean ± standard deviation of the mean, and where relevant with (min, max). All outcomes were inspected for normal distribution using the Kolmogorov-Smirnov test, prior to selection of appropriate statistical tests. The two tailed Mann–Whitney test was used to compare the average weekly training duration in patients with severe impairment of arm function against patients with moderate to mild impairment. A one-way repeated measures ANOVA was used to assess differences over time in training duration, clinical scores and automated assessments, in case of normally distributed data. Otherwise, the non-parametric Friedman test was used. Correlation analysis was used to examine the relationship between clinical assessments and ArmeoSenso-based assessments. Results were considered significant at *p* < 0.05.

### Patient characteristics

Eleven patients were recruited in the University Hospital Zurich (for details see Table 1). In parallel to the study, all patients except for one received physical therapy, on average 3.9 sessions/week, corresponding to approximately 155 min/week (estimated duration of 40 min per therapy session). Only one patient reported that he had no prior experience in using computers, and 8 out of 11 patients reported that they had never played computer games before.

**Table 1:** Baseline characteristics

	Mean ± SD^a^ (min, max)
N	11
Male	5
Right side affected	8
Age, y	60 ± 11.5 (min 42, max 79)
Months post stroke	27 ± 31.5 (min 4, max 118)
NIHSS^b^	3.3 ± 1.2 (min 1, max 5)
mRS^c^	1.9 ± 0.1 (min 1, max 3)
FMA-UE ^c^	35.1 ± 19.9 (min 11, max 60)
WMFT ^d^	52 ± 39 (min 16, max 70)

^a^Standard deviation

^b^National Institutes of Health Stroke Scale (0–42 points)

^c^modified Rankin Scale (0–6 points)

^c^Fugl-Meyer Assessment - Upper Extremity (0–66 points)

^d^Wolf Motor Function Test (0–75 points)

## Results

### Safety and system usage

**Fig. 2:**
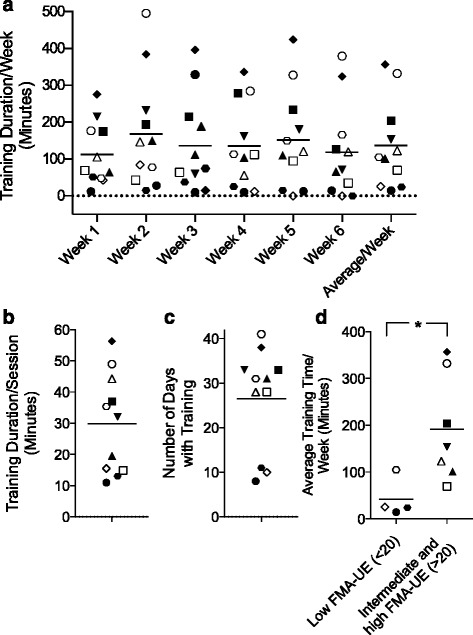
System Usage: **a**-**d**: Each symbol represents one patient. **a**: Weekly training duration for weeks 1–6 and average weekly training duration for each patient. **b**: Training duration per session. **c**: Number of days with training. Horizontal lines indicate averages. **d**: Average weekly training duration in patients with low (<20 points) Fugl-Meyer Assessment Upper Extremity (FMA-UE) and intermediate to high (>20 points) FMA-UE score. * indicates significant differences in usage

All subjects were able to use the system without supervision at their homes and there were no patient-reported adverse events. On average, patients used the system on 26.5 ± 11.5 days (min 8, max 41) out of 42 days (Fig. 2c), corresponding to 4.4 days with training per week. The average training duration per week was 137 ± 120 min (min 15, max 357). The weekly training duration did not change over the course of six weeks (one-way repeated measures ANOVA: *p* = 0.146, F = 1.912, Fig. 2a). According to the patient interviews, 8/11 patients would have liked to continue training with the system and the perceived therapy efficacy was high, with 8/11 patients stating that the trial improved their arm function. Further, 9/11 of patients found the system to be motivating. The two patients who replied negatively were also within the group of those 3/11 patients not stating a desire to continue training with the system. Further, these three patients had a significantly (Wilcoxon rank-sum test, *p* = 0.049) lower initial FMA-UE score (16 ± 7.8) compared to the other eight patients (FMA-UE 43 ± 18.3). These patients also trained less (85 min/w versus 177 min/w), but the difference was not significant (Wilcoxon rank-sum test, p = 0.38).

The average training (gaming) duration per session was 30 ± 16 min (min 11, max 56) (Fig. 2b). The average number of successful arm movements during gaming with Meteors and Slingshot, a measure for training intensity, was 387 ± 522 movements per session (min 40, max 1486). Patients with severe impairment of arm function (FMA-UE ≤ 20, *N* = 4) used ArmeoSenso significantly less (42 ± 42 min/week) than those with moderate and mild arm impairment (FMA-UE > 20, *N* = 7, 191 ± 113 min/week, *p* = 0.024, Fig. 2d).

The average setup duration per therapy session was 4 ± 2 min. The combined average time to complete all assessments for a therapy session was 4 ± 1 min. On average, patients spent 79 % of a therapy session with actual training, i.e. playing either the Meteors or Slingshot therapy game.

### Changes in arm function

**Fig. 3:**
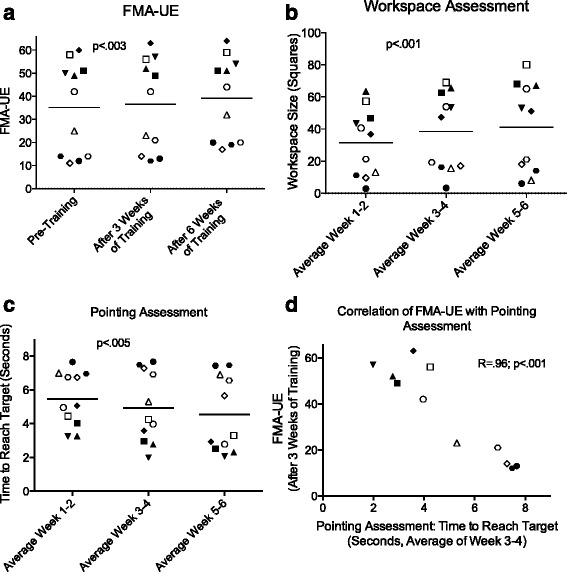
Arm Function Assessments: **a**-**d**: Each symbol represents one patient. **a**-**c**: Horizontal bar = average. **a**: Fugl-Meyer Assessment Upper Extremity (FMA-UE) shows significant improvement after six weeks of therapy. **b**-**d**: ArmeoSenso-based Assessments. In one instance, a patient did not use the system during a block of two weeks. Here, the previous value was carried forward. **b**: Arm Workspace Assessment. The workspace is reported as squares, i.e. relative units for the covered workspace and shows significant improvement after six weeks. **c**: Pointing Task Assessment. The average time to reach targets improves significantly. **d**: Significant correlation between clinical assessment (Fugl-Meyer assessment after 3 weeks of training) and ArmeoSenso assessment (time to reach target, average of training week 3–4, **c**)

Patients showed a significant improvement in the FMA-UE from 35.1 ± 19.9 points to 39.2 ± 17.9 points after 6 weeks, which represents an average improvement of 4.1 ± 2.5 points (one way repeated measures ANOVA: *p* = 0.003, F = 8.701, Fig. 3a). The changes seen in the WMFT were small and not significant (improvement of +1.2 points after six weeks, Friedman-test: *p* = 0.552).

The automated assessments performed at each training session were plotted as two-weekly averages for comparison to clinical scores. The workspace of the affected arm in the transverse plane, as documented by the automated workspace assessment, improved significantly by 31 % between the first two weeks (31.5 ± 20.8 squares) and the last two weeks (40.8 ± 28 squares; one way ANOVA: *p* = 0.008, F = 9.280, Fig. 3b). In the pointing task, the number of targets (out of 9) reached within 8 s improved significantly from 4.4 ± 2.8 in the first two weeks to 5.9 ± 3.1 in the last two weeks (Friedman-test: *p* < 0.001, F = 13.780, data not shown). The average time to reach the targets decreased significantly by 19 %, from 5.4 ± 1.6 s in the first two weeks to 4.5 ± 2.2 s in the last two weeks (one-way ANOVA: *p* = 0.005, F = 7.17, Fig. 3c).

The FMA-UE scores correlated significantly with all three metrics of the automated assessments (number of workspace voxels r = 0.91, *p* < 0.001, number of reached targets r = 0.96, *p* < 0.001, time to reach target r = 0.92, *p* < 0.001, the latter is shown in Fig. 3d).

The therapy dose (i.e. total training duration with ArmeoSenso) did not correlate with the changes over six weeks in the clinical assessments (r = −0.3, *p* = 0.370 for the FMA-UE, and r = −0.083, *p* = 0.809 for the WMFT, data not shown).

### Kinematic analysis

**Fig. 4:**
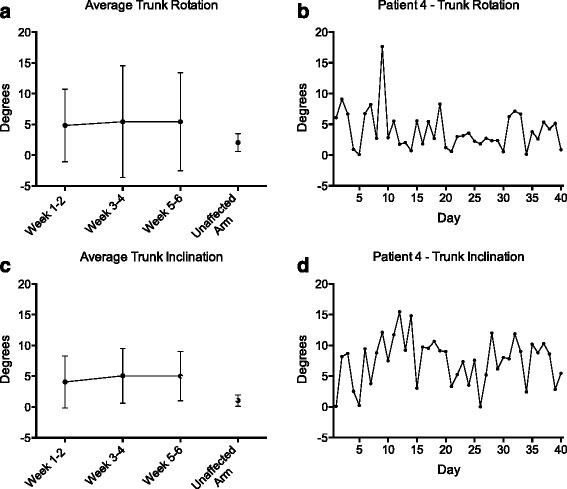
Trunk Movement during Pointing. Trunk rotation (**a**, **b**) and inclination (**c**, **d**) (two-weekly average) during pointing movements in the pointing task assessment for one specific target. For comparison, the values of 10 pointing movements performed with the unaffected limb are plotted (*N* = 8). **b** + **d**: To demonstrate the high inter-session variability of trunk rotation and inclination during pointing movements, a complete dataset of one patient (impaired side) is plotted for the same target. Error bars: standard deviation

Trunk angle analysis during the pointing assessment shows that patients moved their trunk significantly more when reaching with their impaired arm compared to reaching with their unaffected arm (Fig. 4). These trunk movements occurred with a higher variability in the impaired side, as demonstrated by high standard deviations. The average absolute trunk rotation did not change significantly between the first and the last two weeks (one-way ANOVA, p = 0.531, F = 0.415) but was significantly higher when compared to reaching movements with the unaffected arm (one-way ANOVA, *p* = 0.030, F = 5.859). The same was observed for the average trunk inclination (for target 6), which did not change significantly between the first and the last two weeks (one-way ANOVA, *p* =0.208, F = 1.757) but was significantly higher compared to the sessions with the unaffected arm (one-way ANOVA, *p* < 0.001, F =24.968). An example of the high inter-session variability of trunk angles during all training sessions for one patient (and the same target) is shown in Fig. 4b and d.

## Discussion

This paper presents results of a feasibility study using ArmeoSenso, a novel, wearable sensor-based home therapy system with rehabilitative games for arm training and automated IMU-based assessments of arm function. During a six-week intervention, all stroke patients (*N* = 11) were able to train with ArmeoSenso at home without therapist supervision and with no side effects reported, demonstrating that unsupervised self-directed home therapy using a sensor-based virtual therapy platform is feasible and safe. As automated rehabilitation systems carry the risk of being unsuitable for stroke patients due to their complexity, we placed a high priority on developing a system that was easy to use, with therapy exercises that involved intuitive and meaningful, yet challenging movement tasks [27]. The fact that elderly patients (6/11 were aged > 60 years) and patients without gaming experience (8/11) were able to successfully use the system supports its broad applicability. However, patients with severe impairments of arm function used the system less than those with moderate or mild impairments, suggesting that targeted training systems for this group should be developed, e.g. by addition of gravity support.

The therapy dose of 137 min per week (min/week) on average, with training sessions on 4.4 days per week, is promising. Despite the lack of any external therapy supervision after the initial setup day, this result compares favorably to other studies on unsupervised therapy in stroke, where doses of 105 min/week were achieved with the “Supervised Care & Rehabilitation Involving Personal Telerobotics” (SCRIPT) hand orthosis [16, 28], 85 min/week with the “home-based Computer Assisted Arm Rehabilitation” (hCAAR) actuated joystick [7] or 31 min/week with the Virtual Glove upper-limb rehabilitation system [17]. Higher training doses of 214 min/week were achieved with the "Elinor" home therapy system [13], but mandatory weekly hospital visits might have influenced patient compliance. The average training intensity, which was 387 successful reaching and pointing movements per session, is in the range of another study with self-directed home therapy for subacute stroke patients, where 383 exercise repetitions per session were reported [29], and is much higher than the relatively low intensity typically observed in standard rehabilitation sessions for the upper limb (32 functional upper extremity movements per session [30]). The observed training duration did not decline during the six week intervention, indicating that the motivation to train with ArmeoSenso remained high. Overall system usage and the reported desire to continue training after completion of the study protocol suggest that the therapy could even be applied over longer periods. Training efficiency was also high with patients spending almost 80 % of the time using the ArmeoSenso system with actual rehabilitation training. This compares favorably to training times in routine outpatient therapy [31]. Such high training efficiency might lower the threshold to start a therapy session and thus increase the therapy dose within the available time. The system's permanent availability throughout the day in the subjects’ home without the restriction of clinical schedules is an important advantage over tele-rehabilitation approaches [32, 33]. It would be interesting to know how many patients declined to participate and the reasons they give for this. However, this was not documented in this feasibility study. In two cases, there was insufficient space to set up the system in the patient’s homes, which illustrated the fact that size and footprint is an important design criterion for a home-based rehabilitation system.

In the unsupervised setting used here, research therapist did not see the patient over the course of the therapy. Direct monitoring of performance and progress and external intervention was therefore not possible. This motivated the development of short assessment modules that patients performed on a daily basis. To the best of our knowledge, unsupervised, automated assessments that accompany each training session have not been realized until now. The high correlation found between the automated assessments and clinical assessment scales in arm function is a first step towards confirming their validity. In the future, such unsupervised, automated assessments could alert therapists remotely, e.g. via the Internet, about stagnating or declining performance during home training sessions. Therapy games which do not take into account a patient's individual impairment, as with commercial entertainment systems designed for healthy users, are likely to frustrate patients, potentially jeopardizing motivation and compliance. ArmeoSenso therapy games constantly adapt their difficulty and intensity according to the subjects’ performance, and place targets within or at the border of the reachable workspace [18] to maximize engagement and motivation of the subject.

An important function of a therapist is also to monitor and, if needed, correct the patient’s posture and movements in order to prevent the development of pain or maladaptive motor patterns, such as excessive compensatory trunk movements (inclination and rotation) or excessive shoulder abduction during arm reaching [34]. With systems that do not track joint angles (e.g. Nintendo Wii) or commercial games that are not designed for rehabilitation purposes [11, 35], development of such patterns may go unnoticed. Systems based on the use of cameras (e.g. Kinect [12, 35–37]) or IMUs that reconstruct body posture offer the possibility to detect compensatory movements [38]. The reconstruction algorithm implemented in ArmeoSenso attempted to minimize trunk inclination and rotation by directly suppressing their effects in the virtual environment, i.e. only arm movements relative to the trunk are depicted and used as input for the games and assessments. Despite this effort, patients typically exhibited significantly higher trunk inclination and trunk rotation during reaching movements with their impaired arm than with the unimpaired arm [39]. Nevertheless, trunk movements remained small in most patients, with an average of less than five degrees of trunk inclination or rotation. The extent of trunk movement was highly variable (inter-patient and inter-session), and there was no significant trend over time that would suggest either an increase or a reduction of compensation with the trunk during reaching movements. Providing auditory instructions when excessive trunk movements are detected, simulating the presence of a virtual therapist [38], or using negative visual cues within the therapy game [40], might help to prevent compensatory trunk movements.

The mean gain in FMA-UE was 4.1 points, which is not regarded as clinically relevant, but five out of eleven patients showed a clinically relevant improvement of more than 4.25 points [41]. This is comparable to findings for high-intensity therapy in chronic stroke patients [42]. This improvement was not reflected in the WMFT, which improved marginally by 1.2 points. This is likely due to the lack of hand training by ArmeoSenso; hand function is important for performing the WMFT. The improvement of arm function could be explained by the self-directed training with ArmeoSenso or by the standard rehabilitation therapy that most patients received during the study in addition to the experimental training (155 min per week on average).

## Conclusion

This paper presents the design and feasibility of ArmeoSenso, a wearable sensor-based home therapy system for self-directed rehabilitative arm training after stroke. Our results demonstrate that this home therapy is safe and can provide rehabilitative training in a high dose. The integrated assessments allow daily therapy monitoring, difficulty adaptation and detection of maladaptive motor patterns such as trunk movements during reaching. Clinical effectiveness of ArmeoSenso needs to be investigated in a larger randomized controlled trial.

## Abbreviations

FMA-UE, Fugl-Meyer assessment of the upper extremity; GCP, Good Clinical Practice; IMU, Inertial Measurement Unit; WMFT, Wolf Motor Function Test
